# Pneumatic Displacement of a Dense Sub-inner Limiting Membrane Pre-macular Hemorrhage in Dengue Maculopathy: A Novel Treatment Approach

**DOI:** 10.4274/tjo.galenos.2018.94103

**Published:** 2019-02-28

**Authors:** Ashok Kumar, Vikas Ambiya, Vinod Kumar Baranwal, Amit Arora, Gaurav Kapoor

**Affiliations:** 1 Army College of Medical Sciences, Department of Ophthalmology, New Delhi, India

**Keywords:** Dengue maculopathy, sub-ILM hemorrhage, pneumatic displacement

## Abstract

Sub-inner limiting membrane (ILM) hemorrhage is a rare presenting feature of dengue maculopathy. A 24-year-old man in active military service who was recently treated for dengue hemorrhagic fever presented with sub-ILM bleeding in right eye (dominant eye) with profound diminution of vision. Spectral domain optical coherence tomography and fundus fluorescein angiography confirmed sub-ILM hemorrhage with no evidence of vasculitis/venous occlusion or neovascularization. He refused active surgical management by pars plana vitrectomy and was treated with pneumatic tamponade of C3F8 (100%) gas with prone positioning in order to achieve faster visual recovery. He responded well to treatment with complete visual recovery in 1 week. This case report documents for the first time treatment of sub-ILM hemorrhage in the premacular area with pneumatic tamponade in prone position leading to rapid and complete visual recovery in a patient with dengue maculopathy. This novel approach can be employed for patients who are ineligible for more active surgical management.

## Introduction

Dengue fever is an acute viral infection caused by four closely related dengue viruses (Flavivirus) and transmitted through the bite of infected female *Aedes aegypti* mosquitos.^1^ Clinical manifestations of dengue fever range from benign self-limiting fever, chills, and headache to severe dengue hemorrhagic fever and dengue shock syndrome.^2^ Dengue maculopathy may develop in patients with dengue fever. Common ocular manifestations include retinal hemorrhages, macular edema, yellow spots, optic disc swelling, and retinal vasculitis.^3,4,5^ Marked thrombocytopenia in dengue fever may predispose to severe forms of ocular disease and may present with premacular hemorrhages in rare cases.^6^

There is no proven antiviral treatment or commercially available vaccine for both dengue fever and dengue maculopathy.^7^ The use of steroids has been reported in some cases with variable response. However, good visual outcome may also be the natural course of the disease. Certain professionals with premacular hemorrhage complicating dengue maculopathy will require rapid visual recovery which may not be possible by using steroids or letting the disease take its natural course. We report a case of successful treatment of dense sub-ILM premacular hemorrhage in dengue maculopathy with pneumatic displacement using C3F8 gas in an active military personnel requiring early recovery of vision. In this case we used a novel technique of C3F8 pneumatic tamponade and prone positioning to displace dense sub-ILM hemorrhage with minimal intervention to avoid the potential risk of pars plana vitrectomy surgery in a young patient.

## Case Report

**Figure 1:**
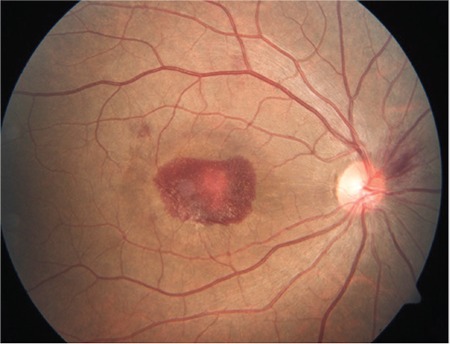
Fundus picture shows premacular hemorrhage about 2 disc diameters in size in the right eye with splinter hemorrhage in the superonasal aspect of the optic disc

**Figure 2:**
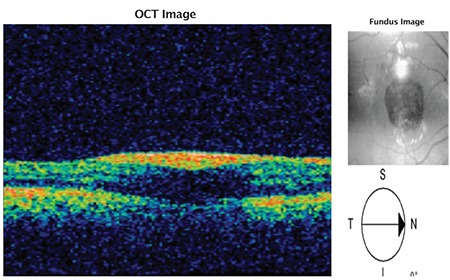
Optical coherence tomography of patient confirming sub-inner limiting membrane hemorrhage in the premacular area

A 24-year-old male active military personnel presented with complaints of profound diminution of vision in his right (dominant) eye of 5 days duration. He had been diagnosed with dengue hemorrhagic fever about 20 days earlier, treated with supportive therapy only without any blood/blood component infusion, and discharged from hospital 10 days earlier. During hospitalization, his lowest platelet count was 40,000 per microliter of blood without any ocular symptoms. On initial examination, his best corrected visual acuity Best-corrected distance visual acuity was 20/400 in right eye and 20/20 in left eye. Anterior segment examination in both eyes was normal. Fundus examination in the right eye revealed premacular hemorrhage about 2 disc diameters (DD) in size occupying the central macula and obscuring underlying details due to a splinter hemorrhage at the superonasal aspect of the disc (Figure 1). There was no evidence of any vasculitis or venous occlusion. Optical coherence tomography (SD-OCT) revealed hemorrhage to be occupying the sub-ILM space, obscuring deeper foveal details (Figure 2). Fundus fluorescein angiography showed blocked fluorescence due to blood in the sub-ILM space, with no evidence of vasculitis or foveolitis. His present systemic work-up was normal and platelet counts revealed mild thrombocytopenia (120,000 per microliter of blood). 

**Figure 3:**
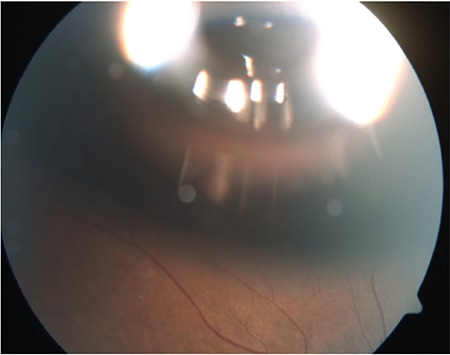
Fundus photograph showing C3F8 gas bubble in the vitreous cavity

**Figure 4:**
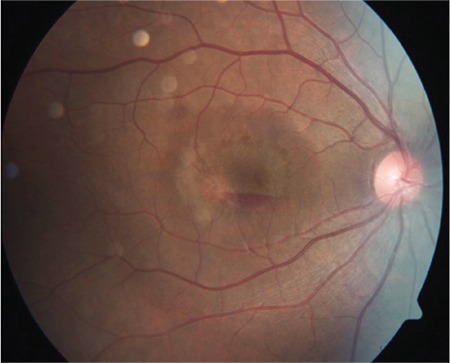
Fundus photograph 3 days post-C3F8 tamponade showing substantial clearing of sub-inner limiting membrane hemorrhage

**Figure 5:**
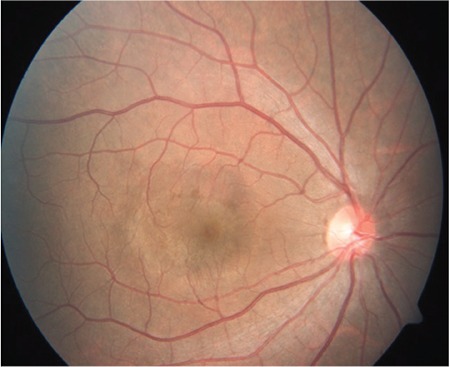
Fundus photograph showing complete clearing of hemorrhage with normal looking posterior pole at 7 days post-C3F8 gas injection

**Figure 6:**
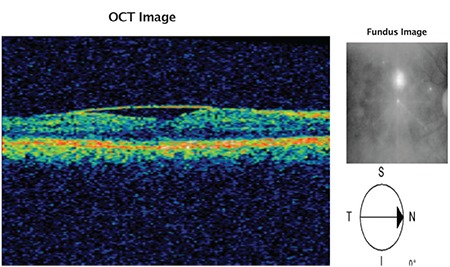
Optical coherence tomography showing normal foveal contour and architecture with overlying inner limiting membrane fold

The patient was informed and counseled about different treatment approaches including “wait-and-watch” for spontaneous recovery, pars plana vitrectomy, and a novel technique of pneumatic displacement with intraocular gas tamponade. The patient did not consent to active surgical management by pars plana vitrectomy. Being in active military service with dominant eye involvement, rapid recovery was warranted, so he was treated with 0.3 ml of C3F8 (100%) injected intravitreally in aseptic conditions followed by paracentesis in the operating theatre with prone positioning (Figure 3). He responded well to treatment with partial displacement and absorption of sub-ILM blood by day 3 post-C3F8 injection (Figure 4) and complete clearing of sub-ILM blood by the end of the first week (Figure 5). OCT showed normal foveal contour with remnants of ILM (Figure 6) seen over the macula with recovery of vision to 20/20 without any metamorphopsia or scotoma.

## Discussion

Dengue maculopathy is the presence of macular swelling, hemorrhages, and yellow spots in the macula due to retinal or choroidal vasculopathy. It can present as macular edema (76.9%), macular hemorrhage (69.2%), foveolitis (28-33.7%), vasculitis, or vascular occlusion.^8^ Other less common dengue-related ocular signs include vitreous hemorrhage and rarely premacular hemorrhages, as seen in our case.^9^ Dengue-related ocular disease is often self-limiting and resolves spontaneously without treatment in 6-8 weeks. Patients with intraretinal vascular or choroidal leakage, signs of active ocular inflammation, and foveal swelling are more likely to benefit from steroid therapy.^10^ Larger studies are needed to validate and justify its usage, as corticosteroid therapy does come with its own side effects.

The prolonged presence of sub-ILM blood as seen in our case may lead to the development of significant epiretinal tissue proliferation with significant visual loss and ocular morbidity.^11^ While pars plana vitrectomy likely has the greatest anatomic success rate, the well-known complications limit immediate use in the majority of situations.^12^Nd:YAG laser hyaloidotomy is another noninvasive method which enables drainage of extensive premacular subhyaloid hemorrhage into the vitreous, facilitates absorption of blood cells, and improves vision within days by clearance of the obstructed macular area.^13^ It has been advocated that hemorrhage less than 3 DD in size should not be subjected to photodisruptive laser for safety reasons. This size helps to increase the cushion effect of the hemorrhage in order to avoid inadvertent retinal damage by the photodisruptive laser.^14^ Iatrogenic foveal or parafoveal macular can also be a potential complication of this procedure.^15^ Our patient was active military personnel with right eye (firing eye) involvement and 2 DD premacular hemorrhage who required rapid visual recovery with no residual visual morbidity, so management with pneumatic displacement was preferred. He underwent minimally invasive intervention in the form of C3F8 tamponade with prone positioning, enabling full visual recovery within 7 days post-intervention. There were no sequelae such as metamorphopsia and persistent central or paracentral scotoma, which were reported in up to 59.5% of 74 affected eyes at 2-year follow-up in a study by Teoh et al.^16^

To the best of our knowledge, this is the first reported case of dengue maculopathy complicated by sub-ILM premacular hemorrhage treated successfully with the minimally invasive novel technique of pneumatic tamponade with prone positioning, resulting in faster and complete visual recovery with no residual adverse effects in a soldier on active duty. However, prospective, randomized trials with large patient numbers will be required to validate the effectiveness and safety of pneumatic tamponade as a treatment modality for premacular sub-ILM hemorrhage in dengue maculopathy.
